# Temporomandibular Disorders and Oral Features in Idiopathic Inflammatory Myopathies (IIMs) Patients: An Observational Study

**DOI:** 10.7150/ijms.45226

**Published:** 2021-07-05

**Authors:** Vito Crincoli, Mariangela Cannavale, Angela Pia Cazzolla, Mario Dioguardi, Maria Grazia Piancino, Mariasevera Di Comite

**Affiliations:** 1 Department of Basic Medical Sciences, Neurosciences and Sensory Organs, “Aldo Moro” University of Bari, Piazza G. Cesare 11, 70124 Bari, Italy.; 2 Department of Surgical Sciences, University of Turin, Italy.; 3 Department of Clinical and Experimental Medicine, University of Foggia, Via Rovelli 50, 71122 Foggia, Italy.

**Keywords:** inflammatory idiopathic myopathies, oral signs and symptoms, temporomandibular disorders

## Abstract

**Aim:** Inflammatory idiopathic myopathies (IIMs) are inflammatory processes affecting skeletal musculature and extramuscular organs. Temporomandibular disorders (TMD) involve jaw muscles and temporomandibular joint. The aim of this observational study was to investigate the prevalence of the main TMD symptoms and signs as well as oral implications in IIM patients.

**Methods:** The study group included 54 patients (42 women and 12 men), 22 of whom affected by dermatomyositis (DM), 29 by polymyositis (PM) and 3 by inclusion body myositis (IBM). A group of 54 patients not affected by this disease, served as CG. Oral and TMD signs and symptoms were evaluated by means of a questionnaire and through clinical examination.

**Results:** About oral symptoms, the study group complained more frequently dysgeusia, with loss of taste or unpleasant taste (*p*<0.0001) and feeling of burning mouth (9.4% versus 0 controls). Xerostomia was more prevalent in the study group respect to the CG (*p*<0.0001). Dysphagia was reported by 48.1% of IIM patients while was absent in CG (*p*<0.0001). About oral signs, cheilitis (*p*<0.05) and oral ulcers (*p*<0.05) were significantly more frequent in CG. As regard to TMD symptoms, arthralgia and tinnitus didn't showed significant differences between the two groups, while neck/shoulders and masticatory muscle pain was significantly more referred in IIM patients than in the CG (*p*<0.05). About TMJ signs, sounds were overlapping in the two groups: click=11.1% in both IIM patients and CG (*p*>0.05), crepitation in 11.1% of IIM and 9.3% of controls (*p*>0.05). No significant difference was detected about deflection (9.3%, *p*>0.05), while deviation was wider in CG (*p*<0.05). Active opening and lateralities showed no significant differences, while endfeel was significantly increased in IIM group for a higher presence of muscular contracture. Bruxism was present only in CG.

**Conclusion:** The data collected from this observational study seem to support the existence of a relationship between the prevalence of TMD symptoms and signs as well as oral features in patients with myositis. A remarkable reduction of salivary flow and dysphagia were more frequent and severe in IIM patients, as well as muscle contracture and myofacial pain evoked by palpation, this result being highly significant.

## Introduction

Inflammatory idiopathic myopathies (IIM) refer to a rare heterogeneous group of acquired muscle diseases, due to still unknown causes and characterized by an inflammatory process affecting skeletal musculature and extramuscular organs 1.

The IIMs can be subgrouped into dermatomyositis (DM), polymyositis (PM) and inclusion body myositis (IBM) 2. The incidence is up to 11 new cases per million inhabitants per year, while the prevalence is 14 out of 100,000 inhabitants 3. The female-to-male ratio is 2:1, though significant differences are present among the subgroups 4.

While PM generally affects individuals aged >18 years and IBM >50 years, DM occurs in all ages. Therefore, in children there is almost exclusively DM, which seems to configure a distinct clinical-pathological form. The most accredited etiopathogenesis is the intervention of exogenous factors capable of triggering an immune and/or autoimmune reaction with a genetically predisposed subject (carrier of the HLA-DRB1 and DRA1 antigens), leading to a muscle damage 5. Triggering factors have been supposed to be those viruses that frequently cause muscular symptoms such as Coxsackie, Echovirus, influenza A and B, hepatitis B and C, Herpes, rubella, Epstein-Barr virus and also retroviruses 6. Non-infectious agents are reputed to be D-penicillamine, L-tryptophan, hypocholesterolomizing agents, interleukin-2, growth hormone, silicone or collagen surgical prostheses, exposure to dust of silica polyvinyl chloride or organic solvents.

The clinical picture of PM and IBM is dominated by muscular weakness, which mainly affects the proximal musculature of the limbs and girdles in a prevalent and symmetrical way. Neck, respiratory and masticatory muscles may be involved, with swallowing impairment and alteration of phonation. Gastrointestinal, pulmonary, renal, articular, and other connective tissue diseases have been described, such as rheumatoid arthritis, systemic sclerosis 7, 8, or rarely Sjögren's syndrome 9. The cutaneous manifestations are characteristic of the DM. A significantly specific sign is the heliotropic rash: purplish coloring of the upper eyelids, sometimes accompanied by edema 10.

Multiple oral manifestations can occur in patients with DM, even severe cases of oral cancer, and sometimes they represent the first sign of the disease 11, 12. Oral features include erythematous mucosal lesions, ulcers, gingival telangiectasias and lingual atrophy, with consequent dysgeusia 13-18. Hyposalivation is present and predispose patients to an increased risk of developing caries, gingival inflammation, periodontal disease, oral infections, especially from fungi of the genus Candida and from staphylococcal superinfection in angular cheilitis 19-21.

About dysphagia in DM, the mechanism is not yet entirely clear but it is thought to be associated with an inefficiency of the laryngeal and subhyoid muscles and an altered functionality of the upper esophageal sphincter 22-27.

Temporomandibular disorders (TMD) are considered a distinct subgroup of musculoskeletal and rheumatoid disorders involving the temporomandibular joint (TMJ) and the jaw muscles. They represent the most important cause of orofacial pain of non-dental origin, with up to 93% of the general population showing at least one TMD symptom or sign. TMD include pain, headaches located in temporal area, masticatory muscle fatigue and limited mandibular movement. These altered functions can lead to difficulties in eating, drinking and swallowing, causing a TMD related oral stage dysphagia (OD) and weight loss. Autoimmune diseases, such as psoriatic or rheumatoid arthritis, Systemic Lupus Erythematosus, Sjögren Syndrome can involve TMJ, leading to a breakdown of the cartilage matrix and bone destruction, with condylar flattening, cortical erosions, osteophytes, sub cortical cysts, osseous sclerosis and gradual decrease in joint space due to granulation. The osseous damage is often responsible of crepitus, a TMD sound noticeable during function, while clicking is usually related to a disc displacement 28-31.

A possible risk factor of TMD is represented by bruxism, a repetitive jaw-muscle activity occurring during sleep (identified as rhythmic or non-rhythmic) and/or awakefulness (with repetitive or sustained dental contact and/or bracing or thrusting of the mandible) 32-34. It becomes pathological when associated with myalgia (due to ischemia and accumulation of metabolic biomarkers in the muscle tissue) and joint pain 17, 35. Hypertrophy of the masticatory muscles is often present.

Currently the literature lacks studies about bruxism in IIMs patients, so this relationship is still unclear. IIMs are characterized by a condition of muscle weakness, caused by a loss of muscle mass and a reduced intrinsic contractility, so this altered function could lead to an unlikely presence of bruxism or, at least, to its marginal role.

On the other side, myalgia during chewing and the positive palpation of most of the stomatognathic muscles confirm that the dermatomyositis/polymyositis seems to play a role in TMD with regards to muscle contracture and soreness, while it is not believed to be involved in joint pain.

Given this background, the aim of this study was to clinically evaluate the prevalence of oral manifestations and main TMJ symptoms and signs in a sample, homogeneous for ethnic origin, of IIM patients on drug therapy, compared with a CG.

## Materials and Methods

This observational study was performed between November 2017 and January 2019 at the School of Dentistry of the University of Bari, Italy, in accordance with the provisions of the Helsinki declaration and approved by the Research Ethics Committee (REC) of Polyclinic of Bari (No. 4900/93348/2015). All patients signed an informed consent.

Fifty-four patients (42 women and 12 men) with DM (22), PM (29) and IBM (3) were recruited in the Complex Operating Unit of Rheumatology, University of Bari, Italy (Table 1).

Inclusion criteria included Caucasian origin and age over 18 years. The exclusion criteria included previous facial trauma, head and neck neoplasia, patients who underwent maxillofacial surgery.

A control group of 54 patients matched by sex (42 women and 12 men), was selected among those presenting at the Dental Clinic for routine oral visit by a randomization list automatically generated (Microsoft Excel) prior to the start of this study.

A patient's medical history was collected, taking into account demographic data (age, marital status, level of education, profession) (Table 2), drugs taken and any comorbidities.

Selective and targeted laboratory tests were not compared with CG, which does not require such investigations (Table 3).

**Figure 1:**
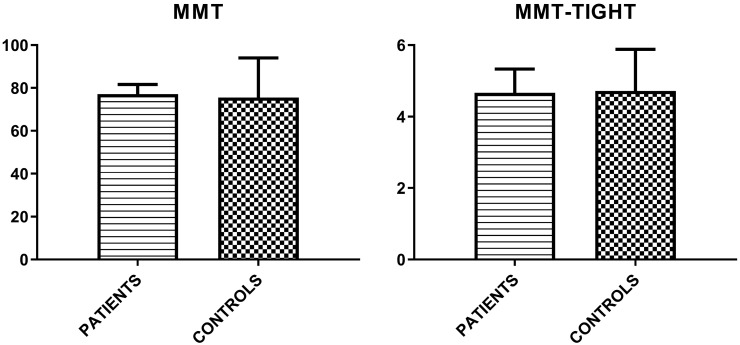
Mean values (± SD) of MMT-8 and MMT-TIGHT evaluations in patient group and controls.

Finally, MMT-8 and MMT-TIGHT evaluations were assessed (Figure 1).

A single practitioner assessed both orofacial manifestations and TMJ features through an anamnestic questionnaire and a clinical examination. Symptoms and signs of the study group were compared to those present in the CG.

### Patients' history

The history questionnaire was used for a dual evaluation: a) Oral symptoms; b) TMD symptoms.

#### Oral symptoms

**(i) Xerostomia:** it is a complaint of dryness of oral cavity. It is classified as true xerostomia, due to malfunction of the salivary glands, or pseudo xerostomia (xerostomia symptomatica), in the course of which the patient has a subjective impression of dry mouth despite a normal secretory function. Xerostomia affects mostly menopausal women and individuals above 65 years of age. It is associated to discomfort in activities such as eating, speaking, swallowing and wearing dentures. Xerostomia can cause a high caries rate and an increased incidence of Candida infection, promoting dental plaque accumulation and periodontal disease 36-38.

**(ii) Dysgeusia:** defined a distorted gustatory perception, often due to a zinc deficiency or to an obstruction of selective taste receptors in taste cells, that can be induced by an autoimmune mechanism. It is an inability to discriminate all the basic tastes (total dysgeusia) or a limited number of basic tastes (partial dysgeusia). Patients report that they perceive bitter, sour, or metallic flavors 39, 40.

**(iii) Stomatodynia:** a burning sensation in the mouth, especially on the tongue, associated with hurtful sensation or pain, even though oral mucosa appears clinically normal. It is often accompanied by other sensory disorders, such as xerostomia and dysgeusia 41, 42.

**(iv) Dysphagia:** it may be complained by the patient as a sensor and/or motor difficulty in the passage of solids from the mouth to the esophagus, probably due to bad training of the bolus, as a result of the reduction in salivary flow or tooth loss. All the above mentioned data were collected as categorical.

#### TMD Symptoms (TMDs)

The presence/absence of the following complaints (TMDs) was recorded: (i) masticatory muscle pain; (ii) tenderness or stiffness in the neck and upper shoulders; (iii) muscle pain during use of the jaw (chewing, etc.); (iv) tenderness or pain in the joint area (arthralgia); (v) difficulty in opening the mouth, a feeling that the jaw was stuck or locked; (vi) less common symptoms, such as dizziness, earache and tinnitus; (vii) headaches located in temporal area 43.

Headache attributed to TMD is usually most prominent in the temporal region, preauricular area of the face and/or masseter muscle. Diagnostic criteria are: (i) Clinical evidence of TMD; (ii) Evidence of causation demonstrated by at least two of the following criteria:

- The headache develops in temporal relation to the onset of the TMD;
- The headache is aggravated by jaw motion (e.g. chewing, bruxism);
- The headache is provoked on physical examination by temporalis muscle palpation and/or passive movement of the jaw;
- The headache improves or disappears as the TMJ disorder improves or resolves.

If the headache is unilateral, it is located ipsilaterally to the side of the TMD, while it is bilateral when the underlying pathology involves both temporomandibular regions 44.

In detail, patients were asked if they had pain in the face, jaw, temple and ear and if this facial pain was persistent, recurrent or was it only a one-time problem. They selected the intensity of perceived current pain on a 100 mm Numeric Pain Rating Scale (NPRS) from 0 to 10. They also referred how much, in the past six months, had facial pain changed their ability to work and to take part in recreational, social and family activities. Through the questionnaire, patients informed the physician about the past or current difficulty in opening the mouth and if this limitation in jaw opening was severe enough to interfere with their ability to eat and swallow. The presence of TMJ sounds (click, pop or crepitation when chewing, opening or closing the mouth) was recorded, as well as tenderness or pain in the joint area (arthralgia), earache, tinnitus and headaches. Also the presence/absence of systemic diseases (e.g. rheumatoid arthritis, Lupus erythematosus), or injuries was assessed.

### Clinical examination

A clinical examination was performed, leaving out I and II level radiological investigations, thus eliminating any type of biological risk for the patient. The exam included a part aimed at the research of oral manifestations and an inherent part finalized to detect the main signs of temporomandibular dysfunction. A single operator conducted the procedure, in order to reduce the bias.

#### Oral signs

The presence/absence of a wide spectrum of signs was taken into account:

- Oral ulcers: presence of oval or roundish sores inside the mouth;
- Petechiae: small (< 3mm) red or brown pinpoint lesions not blanching on pressure, caused by a minor bleed from broken blood vessels, localized more frequently on the hard and soft palate;
- Erythema: reddish and inflamed area on buccal mucosa;
- Cheilitis of lower lip: inflammation state, characterized by redness, swelling and ulcers on lower lip;
- Other oral characteristics analyzed were the integrity of the dental arches or presence of partial or total edentulism and presence/absence of prostheses (mobile, fixed or both).

#### Muscle pain

An important part of the clinical examination of TMD is the assessment of deep pain sensitivity in muscles and joints using manual palpation 45.

A single physician applied a digital pressure on neck and masticatory muscles on the right and left side separately. Prior to palpation, finger pressure was calibrated using an algometer, in order to standardize the pressure 46.

As recommended by the DC/TMD, the digital palpation was carried out with a pressure of 1.0 kgf to the extraoral sites and approximately 0.5 kgf to the intraoral sites for 2 seconds for the diagnosis of myalgia, increasing the duration up to 5 seconds for the diagnosis of referred pain 34, 47.

Anterior, middle and posterior temporalis, superficial masseter, internal pterygoid, digastric, trapezius, sternocleidomastoid and subhyoid muscles were examined extraorally, while external pterygoid, deep masseter and mylohyoid muscles were palpated intraorally. Flat palpation (e.g. on temporalis muscle) or pincher palpation (e.g. on masseter, trapezius, and sternocleidomastoid muscles) were performed. Patients were asked to select the intensity of perceived pain for each muscle according to the following scale: 0= No Pain or just pressure, 1= Mild Pain, 2= Moderate Pain, 3= Severe Pain.

In addition, the condylar lateral pole was palpated to assess the presence/absence of arthralgia, one of the most common pain-related TMD conditions. The lateral pole was identified by placing the index finger just anterior to the tragus of the ear and on the skin overlying TMJ.

A pressure of 1.0 kgf was applied around the lateral pole of the condyle in the protruded position of the mandible for 5 seconds, while a 0.5-kg stimulus was applicated for 2 seconds at the lateral pole with the mandible in a relaxed position 45, 48-50.

#### TMD signs

##### Sounds (TMJs)

The presence/absence of TMJ sounds was evaluated. They are perceived by placing the fingertips on the lateral surface of the condyle on each side separately, during the opening and closing movements of the mandible. Clicking is considered a net, loud joint sound of short duration. It can be single or reciprocal, early or late. Crepitation is a multiple, rough, gravel-like sounds described as grating.

##### Bruxism (BRUX)

It is a repetitive jaw muscle activity, involving central neurological circuits, in particular the dopaminergic transmission, and is characterized by clenching, grinding and/or by bracing or thrusting of the mandible. It is possible to distinguish sleep bruxism (SB) from awake one (AB).

It becomes pathological when associated with myalgia (due to ischemia and accumulation of catabolites in the muscle tissue) and joint pain 17, 35.

Intraoral signs related to bruxism can be: (i) buccal occlusal line; (ii) dental mold on the tongue edges; or, (iii) presence of masticatory muscle hypertrophy. Sometimes, however, even an impaired chewing activity, such as swallowing, can be responsible of these features. In addition: (iv) wear facets, with loss of vertical dimension of occlusion; (v) dental mobility; (vi) failures of dental restorations and/or prosthodontic rehabilitations can be indicative of a probable sleep/awake bruxism. The definite diagnosis of bruxism is given by the use of electromyography, which records level and duration of EMG masticatory activity.

Non-instrumental approaches for assessing bruxism, used in the present work, included self-report (medical history, questionnaires) and clinical inspection.

About SB, patients were asked if they had been told, or had noticed themselves, that they grind their teeth or clench their jaws when they sleep. However, most subjects were unaware of this habit, as it develops unconsciously, and the partner's history was often clarifying. To assess AB, patient were likewise asked whether they grind their teeth or clench their jaws during the day. A positive self-report was considered suggestive of possible bruxism 32, 33, 51, 52.

##### Opening derangement (OD)

In a healthy masticatory system, the opening path taken on the frontal plane by the jaw is straight. Qualitative changes in the opening route are classified as: (i) Deviation: any displacement of the jaw from the midline during the opening that disappears continuing the opening (return to the midline), (ii) Deflection: any displacement of the jaw from the median line that does not disappear in maximum opening and increases by continuing the opening.

##### Restricted movements (RM)

**1) Reduced opening:** in a healthy masticatory system, the mouth opens between 40-60 mm, taking into account the overbite. A mandibular movement is considered reduced when the distance between the incisal margins is <40 mm.

**2) End-feel:** this parameter assesses the quality of movement perceived by the examiner at the end of the passive range-of-motion and is conditioned by the structures that are limiting the movement. It is tested by placing the thumb and the index fingers between patient's upper and lower incisors at the maximal active mouth opening and applying a downward force to passively increase the incisal distance. A soft endfeel and increased mouth opening suggests muscle-caused restriction, while a hard and not increased opening is more likely to be associated with intracapsular sources (e.g. disc displacement without reduction) 53.

Both the maximal active and the passive openings were measured using a caliper or ruler. End-feel was recorded as “positive” when it was above the 2 mm. - the physiological stretching of the ligaments (joint play) - allowing the extent of a paucisymptomatic muscle contracture to be evaluated and compared not as a subjective complaint, but in numerical terms.

**3) Lateral excursion:** right and left lateral shifts were recorded when the distance from upper to lower median line was <8 mm.

**4) Mandibular protrusion:** mean values range between 7 and 10 mm. It was recorded when <7 mm.

### Statistical Analysis

The prevalence of signs and symptoms was analyzed in all the groups described above. The categorical data were expressed as numbers and percentages and compared using Chi-squared test (χ^2^) or Fisher's exact test. The continuous data were reported as mean values and standard deviation (SD) and then compared using the unpaired Student's T test. In all comparisons, values with *p*<0.05 were considered statistically significant.

A preliminary analysis on the reliability of the used set of scale was carried out through *Cronbach'*s *alpha* coefficient. Pooling together patients and control group, computation of such a coefficient was first performed on each of the two sets of items identifying symptoms and signs, and then on the union of these two sets.

Statistical analyses were performed using Prism (GraphPad software, version 6.0, San Diego, California) and R (v. 3.6.1 2019).

## Results

### Demographic Data

The demographic data cannot be used as discriminators of the pathology and do not show significant differences between the two groups.

### Comorbidity

About comorbidities affecting the two groups, the most significant differences concern pathologies such as osteopenia/osteoporosis, thyroid dysfunctions, blood, cardiac, hepatic and gastrointestinal diseases. There are no significant statistical tests for pathologies such as hypovitaminosis, kidney diseases, dyslipidemias, diabetes, lung diseases, hypertension, nervous system diseases and valvular diseases. Lymphadenopathy is absent in both groups (Table 4).

The presence/absence of characteristic aspects of myositis such as Raynaud phenomenon, skin lesions, interstitial lung disease (ILD) and arthritis were assessed (Table 5).

### Pharmacological therapy

Drugs usually given in the myositis sample, such as corticosteroids (68.5%), methotrexate (31.5%), azathioprine (29.6%), mycophenolate (18.5%), immunoglobulin (13.0%) and rituximab (9.3%) are usually absent in CG (Table 6).

No statistical test has proved to be significant about bisphosphonates, diuretics, opioid analgesics, antidepressants, antidiabetics, asa, benzodiazepines, cyclophosphamide, cyclosporine, hydroxychloroquine, statins, tocilizumab and vasodilators. For muscle relaxants and fans/antirheumatics, differences have been detected (Table 7).

#### Oral symptoms

The assessment of referred oral symptoms, obtained from written responses to the questionnaire, revealed *s*tatistically significant differences between the two groups for each oral symptom considered*,* as listed in Table 8.

#### TMJ symptoms

Tinnitus, arthralgia and temporal headache do not show significant differences between the two groups, considering these data as categorical (absence/presence of pain) (Table 9a), while mean values of perceived pain intensity are significantly higher in patient group as regard to headache localized in temporal area (Table 9b).

#### Muscular symptoms

Neck/shoulders and masticatory muscle pain is significantly more present in the group of patients with myositis than in the CG. These categorical data were analyzed using the χ^2^ test (Table 10a).

Pain intensity of masticatory and neck/shoulder muscles is higher in study group (Table 10b).

**Figure 2:**
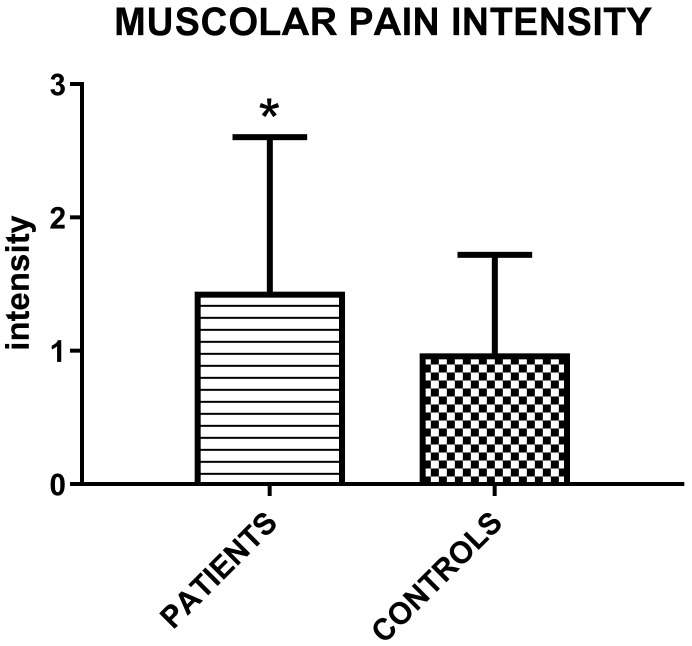
Mean values (±SD) of intensity of perceived myofascial pain under palpation in patient groups and controls according to the following scale: 0= No Pain or just pressure, 1= Mild Pain, 2 = Moderate Pain, 3= Severe Pain (Unpaired T-test; ***p*=0.015**).

The muscolar palpation reveals a higher intensity of perceived myofascial pain in patients group (Figure 2).

#### Oral signs

Erythema showed no statistical significance between the two groups.

The ulcers were analyzed using the Fisher exact test while the cheilitis of the lower lip the Fisher's exact test, both appearing with greater prevalence in the CG (Table 11).

No other manifestations, such as Candidiasis or petechiae were detected in either groups.

#### Oral status

The oral status analyzed the presence/absence of partial or total edentulism. It was prevalent in the group of patients with myositis (24.1%), while, in CG, no patient was edentulous. This data were analyzed using the χ^2^ test (Table 12).

### TMJ signs

Clicks in opening, mutual click, crackling and snap were evaluated. These last two data did not show a statistically significant difference, while the opening click and the mutual click occurred with greater prevalence in CG (Table 13).

### Amplitude of movements

**Figure 3:**
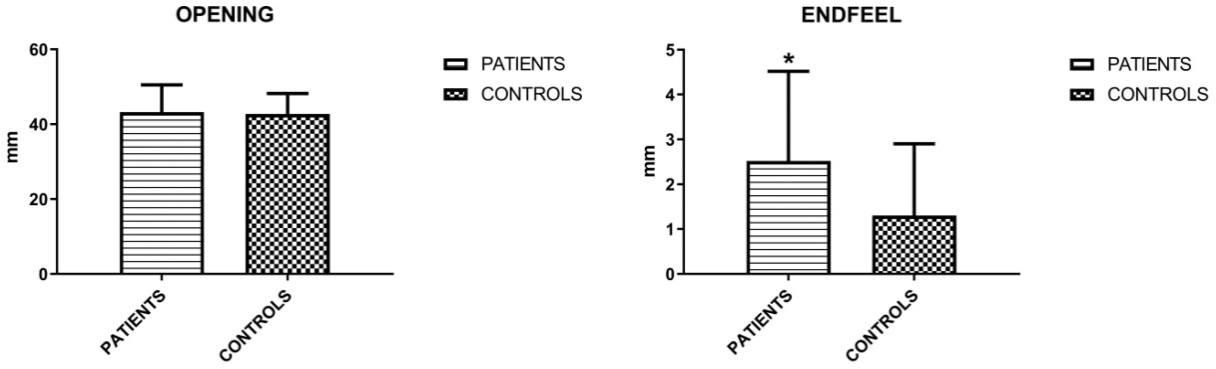
Mean values (±SD) of active opening and endfeel (Unpaired T-test).

**Figure 4:**
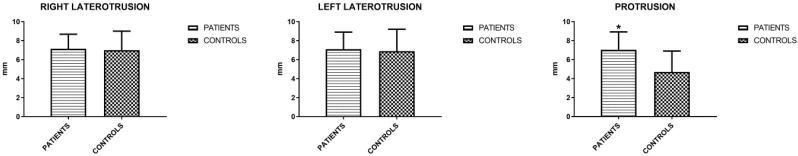
Mean values (±SD) of right and left laterality and protrusion (Unpaired T-test).

The mandibular movements of active and passive opening (endfeel), right and left laterality and protrusion were measured. Active opening and lateralities showed no significant differences, unlike protrusion and endfeel (Figures 3 and 4). Considering this last parameter to be positive for values > 2 mm, the result is indicative for the symptoms of fatigue and muscle pain.

### Quality of Opening

Also a qualitative assessment of mandibular kinematic impairment was carried out. Deviation was wider in CG, while deflection, although more present in the study group, did not show statistical significance with any of the tests applied (Table 14).

### Bruxism

Bruxism, analyzed by Fisher's exact test, results present only in CG. As regards to related oral signs, wear facets and frictional hyperkeratosis (*morsicatio buccarum*) showed no significant differences between the 2 groups; tongue indentations were analyzed with the χ^2^ test and occurred in 7 patients with myositis and in 18 of CG (Table 15), with a significant difference.

### Signs and Symptoms correlation

*Cronbach'*s *alpha* values are listed in the table below, together with the corresponding 95% bootstrap confidence intervals (Table 16).

As it can be easily seen, *Cronbach'*s *alpha* values highlight a strong intercorrelation within each set (signs and symptoms), denoting then that variables are closely related as a group. Furthermore it must be pointed out that the pooled *Cronbach'*s *alpha* reveals a quite high reliability of the used set of scale, being the internal consistency expressed by a value of the covariance equivalent to about 74% of the total variability.

### Dermatomyositis (DM) patients versus control

When performing the same assessments limited to DM patients, oral symptoms were present in 17 patients with DM (77.1%) and 33 controls (61.1%); TMJ symptoms were present in 18 patients with DM (81.8%) and 40 controls (74.07%); muscle symptoms were present in 18 patients with DM (81.8%) and 40 controls (74.07%); oral signs were present in 9 DM patients (40.9%) and 33 control (61.11%) (Table 17).

### Polymyositis (PM) patients versus control

As regards to PM patients, oral symptoms were present in 19 patients with PM (65.5%) and 33 controls (61.1%); TMJ symptoms were present in 23 patients with PM (79.3%) and 40 controls (74.07%); muscle symptoms were present in 19 patients with PM (65.5%) and 40 controls (74.07%); oral signs were present in 12 PM patients (41.37%) and 33 control (61.11%) (Table 18).

### Inclusion body myositis (IBM) patients versus control

As regards IBM patients, oral symptoms were present in 1 patient with IBM (33.3%) and 33 controls (61.1%); TMJ symptoms were present in 3 patients with PM (100%) and 40 controls (74.07%); muscle symptoms were present in 3 patients with PM (100%) and 40 controls (74.07%) and oral signs were present in 3 PM patients (100%) and 33 control (61.11%) (Table 19).

### Dermatomyositis (DM) patients versus polymyositis (DM) versus inclusion body myositis (IBM)

As regards oral symptoms, 17 patients with DM (77.1%), 19 patients with PM (65.5%) and 1 patient with IBM (33.3%) had oral symptoms.

As regards TMJ symptoms, 18 patients with DM (81.8%) 23 patients with PM (79.3%) and 3 patients with PM (100%) had TMJ symptoms.

As regards muscle symptoms, 18 patients with DM (81.8%) 19 patients with PM (65.5%) and 3 patients with PM (100%) had muscle symptoms.

As regards oral signs, 9 DM patients (40.9%) 12 PM patients (41.37%) and 3 PM patients (100%) had oral signs.

## Discussion

Idiopathic inflammatory myopathies (IIM) are a rare, multisystemic disease that can affect a variety of tissues such as skeletal muscle, skin, gastrointestinal tract, lungs, kidneys, eyes, heart, testicles and small joints. The diagnosis of IIM is based on the following criteria: (i) symmetrical weakness of proximal muscles; (ii) muscle biopsy showing features of inflammatory myositis (iii) elevation of serum skeletal muscle enzymes (e.g. CK, LDH); (iv) electromyographic findings (fibrillation in acute phase, low voltage polyphasic potentials); (v) dermatological features (DM rash, including heliotrope and Gottron's papules) 54-56. Testing for myositis specific autoantibodies (MSAs) and myositis-associated autoantibodies (MAAs) can further identify clinical subtype, inform the requirement for further investigations such as MRI, CT scan, echocardiogram and ECG, in order to predict treatment response 57.

The most common oral mucosal findings are telangiectasia due to capillary dilation and bush-loop formation, gingival erythema and dysphagia 11, 14, 16, which can be initial manifestations of the disease and can be considered an important diagnostic marker.

Dourado et al. reported a case of young patient with lichenoid striae in the both retroalveolar mucosal areas, telangiectasia in the posterior teeth gingiva, presenting also some spontaneous bleeding points, gingival calcinosis, in association with papules and erythematous rash in the distal, proximal and metacarpophalangeal joints, compatible with Gottron's papules 21.

Greer et al. showed a case of generalized severe periodontitis with numerous calcifications within the periodontal ligaments of mandibular and maxillary teeth 20.

In the study of Marton et al. the most prominent sign of the oral mucosa and perioral tissues was the presence of telangiectasia. Increased gingival indices without severe periodontal disease should be considered as an unusual sign of IIM, which is probably caused by the edema and erythema secondary to the changes of the gingival capillaries.

Contrary to the works mentioned above, in the present observational study oral ulcers appeared more prevalently in the CG. In detail, using the Fisher exact test, 16.7% of controls were affected versus 0 patients (*p*<0.001). Other manifestations, such as petechiae, were not detected in either groups.

Dysphagia was reported by 48,1% of IIM patients recruited in this study while was absent in CG (*p*<0,0001). This finding can be due to several factors: (i) an impaired oral status, since partial or total edentulism was present in the group of patients with myositis (13 patients, 24.1%), while no patient was edentulous in CG (*p*<0,0001); (ii) a statistically significant prevalence of both masticatory muscle pain (14 patients, 25,9%, *p*<0,022) and fatigue in IIMs group with positive endfeel, (35 patients, 64,81%, *p*=0,0122), which can lead to an oral stage dysphagia (OD); (iii) a highly significant prevalence of xerostomia complained by IIMs patients (29 patients, 54.7%, *p*<0,0001), associated to discomfort in eating and swallowing 24, 25.

Xerostomia is probably related to the drug therapies for treatment of myositis, which include many xerogenic medications. In the present observational study, 68,5% of IIM patients (*p***<**0,001) took corticosteroids, often in addition to others drugs, such as Fans/antirheumatics (9,3%, *p***<**0,028), mycophenolate (18.5%, *p***<**0,001), azathioprine (29.6%, *p***<**0,001), myorelaxants (14,8%, *p***<**0,002). All the aforementioned drugs were absent in CG. This outcome is associated with an increased frequency of loss of taste or unpleasant taste (22,6% of IMM patients against 3,8% of controls) and feeling of burning mouth (9,4% versus 0 controls).

An interesting finding in patients' history is osteoporosis, affecting 12 IIM patients versus 1 of CG (*p*< 0.001), probably due to a prolonged intake of corticosteroids.

Joint involvement with symmetrical non-erosive polyarthritis affecting small joints of the hands can be present as first manifestation in IIM patients 58. It affects from one-fifth to one-half of the patient and may be misdiagnosed as RA. It is more frequent in patients with antisynthetase syndrome (ASS) (prevalence between 50% and 75%) with clinical manifestations of erosive polyarthritis. Other joints can be involved even if less frequently, usually in seronegative RA patients with interstitial lung disease or with Raynaud's phenomenon 59.

In the literature, few studies have focused on masticatory system and temporomandibular joint alterations in IIM and most informations came from case reports and small cohort studies 60.

In the study of Savioli et al. 18, the reduction of mandibular mobility, specifically the ability to open the mouth in patients with active IIM was observed, reinforcing the possibility that this finding is an additional manifestation of juvenile DM in the masticatory system and a consequence of muscle weakness. In a sample of 34 patients with PM and DM, Marton found TMJ alterations in nine patients, while only one control was complaining of that. Five patients demonstrated mandibular deviation and one had crepitation and clicking during opening of the mouth, five of them complained about sensitivity of the lateral pterygoid muscle at palpation 17. Brennan et al. reported a single documented case of bilateral condylar resorption in a patient with dermatomyositis 61. This clinical observational work has a sample size of 54 IIM patient compared with 54 controls to collect information aimed at analyzing both oral and TMJ signs and symptoms, as well as the involvement of stomatognathic and postural muscles, with a cohort size larger than other investigating studies.

About the recruitment of CG, people referring to the clinic may complain of generic oral disturbances more frequently than global population, so this could be a limitation of the present study.

Another weakness lies in the decision of the ethics committee to allow only a clinical-observational study leaving out I and II level radiological investigations, thus eliminating any type of biological risk for the patient. This clinical approach implies a less accurate diagnosis regarding the presence/absence of condylar arthritis/arthrosis.

As for the clinical data attributable to joint problems, i.e. TMJ arthralgia, joint noises and the quality of joint movement, the results did not show a correlation with dermatomyositis/polymyositis. In detail, arthralgia was complained by 16.7% of IIM patients versus 18.5% of controls (p>0.05). The presence of TMJ sounds, which generally indicate morphological alterations of the bony head or joint damage, was overlapping in the two groups: (i) click=11.1% both in IIM patients and in CG (*p*>0.05); (ii) crepitation was present in 11.1% of IIM and 9.3% of controls (*p*>0.05). About qualitative mouth opening, no statistically significant difference was detected about deflection in IIM patients (9.3%, *p*>0.05), while deviation was wider in CG. All these data suggest a weak TMJ impairment in IIM.

In the present study, a clinical, non-instrumental approach was used to assess bruxism, which appears to be present only in CG, since no IIM patient reported being bruxer (*p*<0.05). This absence could be explained by a condition of muscle weakness that develops frequently in this pathology, traditionally thought to be caused by a loss of muscle mass and an impaired intrinsic contractility.

The outcomes of this investigation could be however affected by errors due to subjective data, having screened awake and sleep bruxism through a questionnaire. Future studies should assess bruxism by means of instrumental measurement strategies like an Ecological Momentary Assessment for AB, and electromyography for SB 62, 63. About muscles, parameters that proved to be significant were: (i) endfeel, considering this last parameter to be positive for values > 2 mm; (ii) pain in the muscles of the neck and shoulders in IIM patients (72.2%, *p*<0.0001), (iii) myalgia during chewing and the positive palpation of most of the stomatognathic muscles. All this confirms fatigue and muscle soreness. The following muscles were evaluated: sternocleidomastoid, trapezius, digastric, mylohyoid, posterior, middle and anterior part of the temporalis muscle, masseter muscle, lateral and medial pterygoid muscles. Except for temporal and lateral pterygoid muscles, pain in all masticatory muscles is significantly more felt in the group of patients with myositis than in the CG (*p*<0.022). From the analysis of the data collected in the present work, such as *Cronbach'*s *alpha* values, it emerges a strong intercorrelation within symptoms and signs.

Dermatomyositis/polymyositis seems to play a role in TMD regarding muscle involvement, an element supported by the greater prevalence of myalgia for muscle palpation and positive endfeel (35 patients, 64.81%, *p*=0.0122), that objectively shows a higher presence of paucisymptomatic muscular contracture.

## Conclusion

The findings collected from the present observational study seem to support the existence of a relationship between the prevalence of TMD symptoms and signs as well as oral features in patients with myositis. Particularly, a remarkable reduction of salivary flow and all its consequences such as dysgeusia and dysphagia were found to be more frequent in patients with IIM when compared to a control group. In addition, muscle contracture and myofacial pain evoked by palpation were more frequent and severe in the study group than in the control one, this result being highly significant.

## Figures and Tables

**Table 1:** Classification and duration of myositis

Classification	Patients
DM, n (%)	22 (40.7%)
PM, n (%)	29 (53.7%)
IBM, n (%)	3 (5.6%)
Disease duration, years (mean and ± SD)	3,7 ± 6,4

**Table 2:** Demographic data in patient groups and controls

Demographic Data	Patients	Controls	χ^2^	*p* value
Mean age ± SD	59 ± 13.4	59 ± 13.4		
**Sex, n (%)**				
Males	12 (22.2%)	11 (20.4%)	0.05	> 0.05
Females	42 (78.8%)	43 (79.6%)		
**Marital status, n (%)**				
Married	41 (75.9%)	35 (64.8%)	3.1	> 0.05
Unmarried	7 (13.0%)	9 (16.7%)		
Widowers	3 (5.6%)	8 (14.8%)		
Divorced	3 (5.6 %)	2 (3.7%)		
**Qualification, n (%)**				
Primary school	12 (22.2%)	13 (24.1%)	8.7	> 0.05
Middle School	21 (38.9%)	9 (16.7%)		
High school	13 (24.1%)	19 (35.2%)		
Graduation	7 (13%)	13 (24.1%)		
**Occupation, n (%)**				
Unemployed	2 (3.7%)	1 (1.9%)	13.5	> 0.05
Housewives	17 (31.5%)	20 (37%)		
Public employees	6 (11.1%)	16 (29.6%)		
Freelancers	2 (3.7%)	5 (9.3%)		
Students	1 (1.9%)	0		
Retirees	20 (37%)	11 (20.4%)		
Workmen/craftsmen	6 (11.1%)	1 (1.9%)		

**Table 3:** Laboratory tests in the patient group

Laboratory examinations	Patients
CPK U/L (mean and ± SD)	312 ± 1134.2
LDH U/L (mean and ± SD)	330,8 ± 142.6
VES mm/h (mean and ± SD)	24,8 ±22.5
Anti-synthetase antibodies, n (%)	42 (77.8%)
Associated myositis antibodies, n (%)	41 (75.9%)

**Table 4:** Comorbidity in patient groups and controls

Comorbidity	Patients	Controls	Test	*p* value
Osteopenia/osteoporosis	12 (22.2%)	1 (1.9%)	χ^2^ =10,5	**0.001**
Thyroid dysfunction	18 (33.3%)	6 (11.1%)	χ^2^ =7,7	**0.005**
Blood diseases	4 (7.4%)	0	Fisher's exact test	**0.049**
Heart diseases	13 (24.1%)	4 (7.5%)	χ^2^ =5,6	**0.017**
Liver diseases	12 (22.2%)	0	χ^2^ =13,5	**0.0002**
Gastrointestinal diseases	7 (13.0%)	0	Fisher's exact test	**0.006**
Hypovitaminosis	4 (7.4%)	1 (1.9%)	Fisher's exact test	> 0.05
Kidney diseases	3 (5.6%)	1 (1.9%)	Fisher's exact test	> 0.05
Dyslipidemia	2 (3.7%)	2 (3.7%)	Fisher's exact test	> 0.05
Diabetes	5 (9.3%)	5 (9.3%)	χ^2^ =0	> 0.05
Lung diseases	3 (5.6%)	0	Fisher's exact test	> 0.05
Hypertension	20 (37.0%)	17 (31.5%)	χ^2^ =0,4	> 0.05
Diseases of the nervous system	4 (7.4%)	1 (1.9%)	Fisher's exact test	> 0.05
Valvulopathy	2 (3.7%)	0	Fisher's exact test	> 0.05
Other comorbidities	30 (55.6%)	9 (16.7%)	χ^2^ =17,6	**0.00002**

**Table 5:** Manifestations of the disease in patient group and controls

Manifestations of pathology	Patients	Controls	χ^2^	*p* value
Raynaud	17 (31.5%)	0	20	**< 0.0001**
Skin lesions	27 (50%)	0	36	**< 0.0001**
ILD	21 (38.9%)	0	26	**< 0.0001**
Arthritis	14 (25.9%)	3 (5.6%)	16	**< 0.0001**

**Table 6:** Drugs for treatment of myositis

Drugs	Patients	Controls	Test	*p* value
Corticosteroids	68.5%	0%	χ^2^ =56	**<0.001**
Methotrexate	31.5%	0%	χ^2^ =20	**<0.001**
Azathioprine	29.6%	0%	χ^2^ =18,8	**<0.001**
Mycophenolate	18.5%	0%	χ^2^ =11	**<0.001**
Immunoglobulin	13.0%	0%	Fisher	** 0.006**
Cyclosporine	3.7%	0%	χ^2^ =2	> 0.05
Hydroxychloroquine	3.7%	0%	Fisher	> 0.05
Cyclophosphamide	1.9%	0%	Fisher	> 0.05
Tocilizumab	3.7%	0%	Fisher	> 0.05
Rituximab	9.3%	0%	Fisher	**0.028**

**Table 7:** Drugs taken in the patient groups and controls

Drugs	Patients	Controls	Test	*p* value
Antihypertensive	29.6%	27,8%	χ^2^ =0,04	> 0.05
Myorelaxants	14.8%	0%	Fisher	**0.002**
Bisphosphonates	11.1%	1.9%	Fisher	> 0.05
Diuretics	9.3%	1.9%	Fisher	> 0.05
Fans/antirheumatics	9.3%	0%	Fisher	**0.028**
Opioid analgesics	0%	1.9%	Fisher	> 0.05
Antidepressants	1.9%	0%	Fisher	> 0.05
Antidiabetic	5.6%	9.3%	Fisher	> 0.05
ASA	7.4%	1.9%	Fisher	> 0.05
BDZ	3.7%	0%	Fisher	> 0.05
Statins	1.9%	5.6%	Fisher	> 0.05
Vasodilators	1.9%	0%	Fisher	> 0.05
Others	81.5%	5.6%	χ^2^ =63	**<0.001**

**Table 8:** Subjective complaints of oral discomfort in patient group and control

Oral symptoms	Patients	Controls	χ^2^	*p* Value
Xerostomia	29 (54.7%)	8 (15.1%)	18.310	**<0.0001**
Dysgeusia	12 (22.6%)	2 (3.8%)	8.230	**<0.0001**
Stomatodynia	5 (9.4%)	0 (0%)	5.248	**<0.0041**
Dysphagia	26 (48.1%)	0 (0%)	34.450	**<0.0001**

**Table 9a:** TMJ symptoms in patient groups and controls

TMJ symptoms	Patients	Controls	χ^2^	*p* value
Tinnitus	21 (38.9%)	13 (24.1%)	3	> 0.05
Arthralgia	9 (16.7%)	10 (18.5%)	0,06	> 0.05
Temporal headache	17 (31.5%)	14 (25.9%)	0,5	> 0.05

**Table 9b:** Mean values and ±SD of Numeric Pain Rating Scale (NPRS) in patient groups and controls (Range values 0-10; Unpaired T-test)

	Patients	Controls	*p* value
Arthralgia	1,04 ±2,52	0,56 ±1,38	< 0.05
Temporal headache	1,86 ±3,15	0,74 ±1,48	**<0.021**

**Table 10a:** Muscle symptoms in patient groups and controls

Muscular symptoms	Patients	Controls	χ^2^	*p* value
Digastric	17 (31.5%)	2 (3.7%)	14	**0.0001**
Deep Masseter	25 (46.3%)	8 (14.8%)	13	**0.0003**
Superficial masseter	25 (46.3%)	14 (25.9%)	5	**0.027**
Mylohyoid	17 (31.5%)	3 (5.6%)	12	**0.0005**
Subhyoid muscles	30 (55.6%)	0	41	**<0.0001**
External pterygoid	34 (63%)	34 (63%)	0	> 0.05
Internal pterygoid	30 (56.6%)	15 (27.8%)	9	**0.0025**
Sternocleidomastoid	22 (40.7%)	9 (16.7%)	8	**0.0056**
Anterior temporal	13 (24.5%)	16 (29.6%)	0,3	> 0.05
Middle temporal	12 (22.2%)	9 (16.7%)	0,5	> 0.05
Posterior temporal	11 (20.4%)	10 (18.5%)	0,05	> 0.05
Trapezius	29 (26.9%)	0	40	**<0.0001**
Neck/shoulder pain	39 (72.2%)	2 (3.7%)	54	**<0.0001**
Masticatory muscle pain	14 (25.9%)	5 (9.3%)	5	**0.022**

**Table 10b:** Mean values and ±SD of Numeric Pain Rating Scale (NPRS) in patient groups and controls (Range values 0-10; Unpaired T-test)

	Patients	Controls	*p* value
Masticatory muscles	0.96 ±2.14	0.20 ±0.68	**0.016**
Neck/shoulder	3.39 ±3.42	0.15 ± 0.79	**> 0.0001**
Muscle pain during function	1.39 ±2.97	1.28 ±1.90	< 0.05

**Table 11:** Oral signs in patient groups and controls

Oral signs	Patients	Controls	Test	*p* value
Erythema	1 (1.9%)	0	Fisher	> 0.05
Ulcers	0	9 (16.7%)	Fisher	**0.001**
Cheilitis	0	4 (7.4%)	Fisher	**0.048**

**Table 12:** Partial or total edentulism in patient and control group

Edentulism			
Patients	Controls	χ^2^	*p*
13 (24.1%)	0	14.8	**0.0001**

**Table 13:** TMJ sounds in patient groups and control

Joint noise	Patients	Controls	Test	*p* value
Click	6 (11.1%)	6 (11.1%)	0	> 0.05
Crackling	6 (11.1%)	5 (9.3%)	0,1	> 0.05
Mutual click	2 (3.7%)	8 (14.8%)	4	**0.0463**
Opening click	7 (13%)	15 (27.8%)	Fisher	**0.0465**

**Table 14:** Qualitative opening in patient groups and controls

Quality of opening	Patients	Controls	χ^2^	*p* value
Deviation	3 (5.6%)	29 (53.7%)	30	**< 0.00001**
Deflection	5 (9.3%)	2 (3.7%)	1,3	> 0.05

**Table 15:** Bruxism in patient groups and controls

	Patients	Controls	TEST	*p* value
Bruxism (S/A)	0	8 (14.8%)	Fisher	**0.0029**
Clenching	16 (29.6%)	19 (35.2%)	χ^2^ = 0.3	> 0.05
Wear facets	18 (33.3%)	19 (35.2%)	χ^2^ = 0.04	> 0.05
Oral frictional hyperkeratosis	11 (20.4%)	9 (16.7%)	χ^2^ = 0.2	> 0.05
Indentations of the tongue	7 (13%)	18 (33.3%)	χ^2^ = 6.2	**0.01**

**Table 16:** Cronbach's alpha values and confidence intervals referred to patients and control group pooled together

Sets of variables	Cronbach's alpha values	95% bootstrap confidence intervals
Symptoms	0.69	0.61-0.71
Signs	0.69	0.60-0.77
Symptoms + signs	0.74	0.68-0.81

**Table 17:** Oral signs and symptoms in DM patients and controls

Dermatomyositis	Patients	Controls
TMJ symptoms	18 (81.8%)	40 (74.07%)
Muscle symptoms	18 (81.8%)	40 (74.07%)
Oral signs	9 (40.9%)	33 (61.11%)

**Table 18:** Oral signs and symptoms in PM patients and controls

Polymyositis	Patients	Controls
TMJ symptoms	23 (79.3%)	40 (74.07%)
Muscle symptoms	19 (65.5%)	40 (74.07%)
Oral signs	12 (41.37%)	33 (61.11%)

**Table 19:** Oral signs and symptoms in IBM patients and controls

Inclusion body myositis	Patients	Controls
TMJ symptoms	3 (100%)	40 (74.07%)
Muscle symptoms	3 (100%)	40 (74.07%)
Oral signs	3 (100%)	33 (61.11%)

## Abbreviations

IIM: Inflammatory idiopathic myopathies
DM: dermatomyositis
PM: polymyositis
IBM: inclusion body myositis
TMD: temporomandibular disorders
TMJ: temporomandibular joint
TMDs: symptoms of temporomandibular disorders
TMJs: sounds of temporomandibular joint
OD: opening derangement
VAS: visual analogue scale
MP: myofascial pain
RM: restricted movements
BRUX: bruxism
SB: sleep bruxism
AB: awake bruxism
CG: control group
MMT: manual muscle testing

## References

[1] Clark KE, Isenberg D (2018). A review of inflammatory idiopathic myopathy focusing on polymyositis. Eur J Neurol.

[2] Lundberg IE (2017). Myositis in 2016: New tools for diagnosis and therapy. Nat RevRheumatol.

[3] Svensson J, Arkema EV, Lundberg IE, Holmqvist M (2017). Incidence and prevalence of idiopathic inflammatory myopathies in Sweden: a nationwide population-based study. Rheumatology (Oxford).

[4] Molberg Ø, Dobloug C (2016). Epidemiology of sporadic inclusion body myositis. Curr Opin Rheumatol.

[5] Koler RA, Montemarano A (2001). Dermatomyositis. Am Fam physician.

[6] Au WY, Trendell-Smith NJ, Ko BH, Tong AC, Wong WS (2010). Oral Epstein-Barr virus-related B-cell lymphoma causing persistent paraneoplastic dermatomyositis after nasopharyngeal and cutaneous carcinomas. Leuk lymphoma.

[7] Simon NG, Noto YI, Zaidman CM (2016). Skeletal muscle imaging in neuromuscular disease. J Clin Neurosci.

[8] Dankó K, Ponyi A, Constantin T, Borgulya G, Szegedi G (2004). Long-term survival of patients with idiopathic inflammatory myopathies according to clinical features: a longitudinal study of 162 cases. Medicine (Baltimore).

[9] Crincoli V, Di Comite M, Guerrieri M, Rotolo RP, Limongelli L, Tempesta A (2018). Orofacial manifestations and temporomandibular disorders of Sjögren syndrome: an observational study. Int J Med Sci.

[10] Muro Y, Sugiura K, Akiyama M (2016). Cutaneous manifestations in dermatomyositis: key clinical and serological features-a comprehensive review. Clin Rev Allergy Immunol.

[11] Healy CM, Tobin AM, Kirby B, Flint SR (2006). Oral lesions as an initial manifestation of dermatomyositis with occult malignancy. Oral Surg Oral Med Oral Pathol Oral Radiol Endod.

[12] Sugiyama T, Nakagawa T, Inui M, Tagawa T (2001). Tongue carcinoma in a young patient with dermatomyositis: a case report and review of the literature. J Oral Maxillofac Surg.

[13] Goncalves LM, Bezerra-Júnior JRS, Gordón-Núñez MA, Liberio SA, De Fátima Vasconcelos Pereira A, Da Cruz MCFN (2011). Oral manifestations as important symptoms for juvenile dermatomyositis early diagnosis: a case report. Int J Paediatr Dent.

[14] Ghali FE, Stein LD, Fine JD, Burkes EJ, McCauliffe DP (1999). Gingival telangiectases: an underappreciated physical sign of juvenile dermatomyositis. Arch Dermatol.

[15] Jurge S, Kuffer R, Scully C, Porter SR (2006). Mucosal disease series. Number VI. Recurrent aphthous stomatitis. Oral dis.

[16] Rider LG, Atkinson JC (2009). Images in clinical medicine. Gingival and periungual vasculopathy of juvenile dermatomyositis. N Engl J Med.

[17] Márton K, Hermann P, Dankó K, Fejérdy P, Madléna M, Nagy G (2005). Evaluation of oral manifestations and masticatory force in patients with polymyositis and dermatomyositis. J Oral Pathol Med.

[18] Savioli C, Silva CA, Fabri GM, Kozu K, Campos LM, Bonfa E (2010). Gingival capillary changes and oral motor weakness in juvenile dermatomyositis. Rheumatology (Oxford).

[19] Barbe AG (2018). Medication-induced xerostomia and hyposalivation in the elderly: culprits, complications, and management. Drugs Aging.

[20] Greer RO, Galbraith CT, Scheidt M, Aubry PL, Glasgow MJ (2016). Dermatomyositis with Calcifications of the Periodontal Ligament: A Rare Oral Finding. Dermatol Case Rep.

[21] Dourado MR, da Silva Filho TJ, Salo T (2017). Oral signs in juvenile dermatomyositis. J Oral Diag.

[22] Raymond MJ, McColloch NL, Hatcher JL (2019). Upper Esophageal Sphincter Dilation for Recalcitrant Dysphagia Secondary to Dermatomyositis. Ear, Nose Throat J.

[23] Mugii N, Hasegawa M, Matsushita T, Hamaguchi Y, Oohata S, Okita H (2016). Oropharyngeal dysphagia in dermatomyositis: associations with clinical and laboratory features including autoantibodies. PLoS One.

[24] Gilheaney Ó, Stassen LF, Walshe M (2018). Prevalence, nature, and management of oral stage dysphagia in adults with temporomandibular joint disorders: findings from an Irish cohort. J Oral Maxillofac Surg.

[25] Gilheaney Ó, Béchet S, Kerr P, Kenny C, Smith S, Kouider R (2018). The prevalence of oral stage dysphagia in adults presenting with temporomandibular disorders: a systematic review and meta-analysis. Acta Odontol Scand.

[26] Ortu E, Giannoni M, Ortu M, Gatto R, Monaco A (2014). Oropharyngeal airway changes after rapid maxillary expansion: the state of the art. Int J Clin Exp Med.

[27] Monaco A, Ortu E, Giannoni M, D'Andrea P, Cattaneo R, Mummolo A (2020). Standard Correction of Vision Worsens EMG Activity of Pericranial Muscles in Chronic TMD Subjects. Pain Res Manag.

[28] Crincoli V, Piancino MG, Iannone F, Errede M, Di Comite M (2020). Temporomandibular Disorders and Oral Features in Systemic Lupus Erythematosus Patients: An Observational Study of Symptoms and Signs. Int J Med Sci.

[29] Crincoli V, Anelli MG, Quercia E, Piancino MG, Di Comite M (2019). Temporomandibular disorders and oral features in early rheumatoid arthritis patients: an observational study. Int J Med Sci.

[30] Crincoli V, Fatone L, Fanelli M, Rotolo RP, Chialà A, Favia G (2016). Orofacial manifestations and temporomandibular disorders of systemic scleroderma: an observational study. Int J Mol Sci.

[31] Crincoli V, Di Comite M, Di Bisceglie MB, Fatone L, Favia G (2015). Temporomandibular disorders in psoriasis patients with and without psoriatic arthritis: an observational study. Int J Med Sci.

[32] Lobbezoo F, Ahlberg J, Raphael K, Wetselaar P, Glaros A, Kato T (2018). International consensus on the assessment of bruxism: Report of a work in progress. J Oral Rehabil.

[33] Osiewicz M, Lobbezoo F, Ciapała B, Pytko-Polończyk J, Manfredini D (2020). Pain predictors in a population of temporomandibular disorders patients. J Clin Med.

[34] Schiffman E, Ohrbach R, Truelove E, Look J, Anderson G, Goulet JP (2014). Diagnostic criteria for temporomandibular disorders (DC/TMD) for clinical and research applications: recommendations of the International RDC/TMD Consortium Network and Orofacial Pain Special Interest Group. J Oral Facial Pain Headache.

[35] Jounger SL, Christidis N, Svensson P, List T, Ernberg M (2017). Increased levels of intramuscular cytokines in patients with jaw muscle pain. J Headache Pain.

[36] Greenspan D (1996). Xerostomia: diagnosis and management. Oncology (Williston Park, NY).

[37] Wiener RC, Wu B, Crout R, Wiener M, Plassman B, Kao E (2010). Hyposalivation and xerostomia in dentate older adults. J Am Dent Assoc.

[38] Tanasiewicz M, Hildebrandt T, Obersztyn I (2016). Xerostomia of Various Etiologies: A Review of the Literature. Adv Clin Exp Med.

[39] Mott AE, Grushka M, Sessle BJ (1993). Diagnosis and management of taste disorders and burning mouth syndrome. Dent. Clin. North Am.

[40] Nakazato Y, Ito Y, Naito S, Tamura N, Shimazu K (2008). Dysgeusia limited to sweet taste in myasthenia gravis. Intern Med.

[41] Fedele S, Fricchione G, Porter S, Mignogna M (2007). Burning mouth syndrome (stomatodynia). QJM: An International Journal of Medicine.

[42] Aravindhan R, Vidyalakshmi S, Kumar MS, Satheesh C, Balasubramanium AM, Prasad VS (2014). Burning mouth syndrome: A review on its diagnostic and therapeutic approach. J Pharm & Bioallied Sci.

[43] Dworkin SF, LeResche L (1992). Research diagnostic criteria for temporomandibular disorders: review, criteria, examinations and specifications, critique. J. Craniomand. Disord.

[44] Headache Classification Committee of the International Headache Society (IHS) The International Classification of Headache Disorders, 3rd edition Cephalalgia. 2018; 38: 1-211.

[45] Serrano-Hernanz G, Futarmal Kothari S, Castrillón E, Álvarez-Méndez AM, Ardizone-García I, Svensson P (2019). Importance of Standardized Palpation of the Human Temporomandibular Joint. J Oral Facial Pain Headache.

[46] Noguchi T, Kashiwagi K, Fukuda K (2020). The effectiveness of stabilization appliance therapy among patients with myalgia. Clin Exp Dent Res.

[47] Conti PCR, Silva RdS, Araujo CdRPd, Rosseti LMN, Yassuda S, Silva ROFd (2011). Effect of experimental chewing on masticatory muscle pain onset. J Appl Oral Sci.

[48] Goulet JP, Clark JT (1990). Clinical TMJ examination methods. J Calif Dent Assoc.

[49] Dworkin SF, LeResche L, DeRouen T, Von Korff M (1990). Assessing clinical signs of temporomandibular disorders: reliability of clinical examiners. J Prosthet Dent.

[50] Cunha CO, Pinto-Fiamengui LMS, Castro ACPC, Lauris JRP, Conti PCR (2014). Determination of a pressure pain threshold cut-off value for the diagnosis of temporomandibular joint arthralgia. J Oral Rehabil.

[51] Lavigne GJ, Khoury S, Abe S, Yamaguchi T, Raphael K (2008). Bruxism physiology and pathology: an overview for clinicians. J Oral Rehabil.

[52] Pietropaoli D, Ortu E, Giannoni M, Cattaneo R, Mummolo A, Monaco A (2019). Alterations in Surface Electromyography Are Associated with Subjective Masticatory Muscle Pain. Pain Res Manag.

[53] Okeson JP (2013). Management of temporomandibular disorders and occlusion. 7th ed. Mosby: Elsevier Inc.

[54] Bohan A, Peter JB Polymyositis and dermatomyositis (first of two parts) N Engl J Med. 1975; 292: 344-7.

[55] Lundberg IE, de Visser M, Werth VP (2018). Classification of myositis. Nat Rev Rheumatol.

[56] Antoine M, Reeves PT, Rohena L, Jones O, Faux B (2018). Fashionably Late: A Case of Delayed Cutaneous Manifestations in Juvenile Dermatomyositis. J Clin Med Res.

[57] Gunawardena H (2017). The clinical features of myositis-associated autoantibodies: a review. Clin Rev Allergy Immunol.

[58] Dalakas MC (2015). Inflammatory muscle diseases. New Engl J Med.

[59] Klein M, Mann H, Vencovský J (2019). Arthritis in Idiopathic Inflammatory Myopathies. Curr Rheumatol Rep.

[60] Sun C, Lee J-H, Yang Y-H, Yu H-H, Wang L-C, Lin Y-T (2015). Juvenile dermatomyositis: a 20-year retrospective analysis of treatment and clinical outcomes. Pediatr Neonatol.

[61] Brennan MT, Patronas NJ, Brahim JS (1999). Bilateral condylar resorption in dermatomyositisA case report. Oral Surg Oral Med Oral Pathol Oral Radiol Endod.

[62] Zani A, Lobbezoo F, Bracci A, Ahlberg J, Manfredini D (2019). Ecological momentary assessment and intervention principles for the study of awake bruxism behaviors, Part 1: general principles and preliminary data on healthy young Italian adults. Front. Neurol.

[63] Osiewicz MA, Lobbezoo F, Bracci A, Ahlberg J, Pytko-Polończyk J, Manfredini D (2019). Ecological Momentary Assessment and Intervention Principles for the study of awake bruxism behaviors, part 2: development of a smartphone application for a multicenter investigation and chronological translation for the Polish version. Front. Neurol.

